# Recombinant l-glutaminase obtained from *Geobacillus thermodenitrificans* DSM-465: characterization and *in silico* elucidation of conserved structural domains†

**DOI:** 10.1039/c8ra04740e

**Published:** 2019-02-01

**Authors:** Luqman Shah, Muhammad Shahid Nadeem, Jalaluddin Azam Khan, Mustafa A. Zeyadi, Mazin A. Zamzami, Kaleemuddin Mohammed

**Affiliations:** Department of Biochemistry, Faculty of Science, King Abdulaziz University Building A 90 Jeddah 21589 Saudi Arabia mhalim@kau.edu.sa luqmanshah89@yahoo.com jjalal@kau.edu.sa mzyadi@kau.edu.sa kaleem_kamran111@yahoo.com mzamzami@kau.edu.sa

## Abstract

Glutaminase (GLS) is an enzyme essential for amino acid metabolism; in particular, it acts as a catalyst in glutaminolysis, a reaction exploited by the malignant cells to meet the nutrient requirements for their accelerated growth and proliferation. *Via* regulating the initial reaction of the glutaminolysis pathway, glutaminase offers an intriguing target for the development of anticancer drugs. In the present study, we produced a recombinant glutaminase from *Geobacillus thermodenitrificans* DSM-465 in *E. coli*. The enzyme was purified to electrophoretic homogeneity, with 40% recovery and 22.36 fold purity. It exhibited a molecular weight of 33 kDa, with an optimum pH and temperature of 9 and 70 °C, respectively. The *K*_M_ value of the purified enzyme was 104 μM for l-glutamine. A 3D model was built for the enzyme using Swiss-Model and subjected to molecular docking with the substrate and potential inhibitors. Moreover, the subject enzyme was compared with the human kidney type GLS-K by ConSurf and TM-align servers for evolutionary conserved residues and structural domains. Despite having less than 40% amino acid identity, the superimposed monomers of both enzymes exhibited ∼94% structural identity. With a positional difference, the active site residues Ser65, Asn117, Glu162, Asn169, Tyr193, Tyr245, and Val263 found in the bacterial enzyme were also conserved in the human GLS-K. Molecular docking results have shown that CB-839 is the best inhibitor for GLS-GT and UPGL00004 is the best inhibitor for GLS-K, as designated by the binding free energy changes, *i.e.* Δ*G* −388.7 kJ mol^−1^ and Δ*G* −375 kJ mol^−1^, respectively. Moreover, six potential inhibitory molecules were ranked according to their binding free energy change values for both enzymes. The information can be used for the *in vivo* anticancer studies.

## Abstract 2

Glutaminase (GLS) is an enzyme essential for amino acid metabolism; in particular, it acts as a catalyst in glutaminolysis, a reaction exploited by the malignant cells to meet the nutrient requirements for their accelerated growth and proliferation.

## Graphical abstract

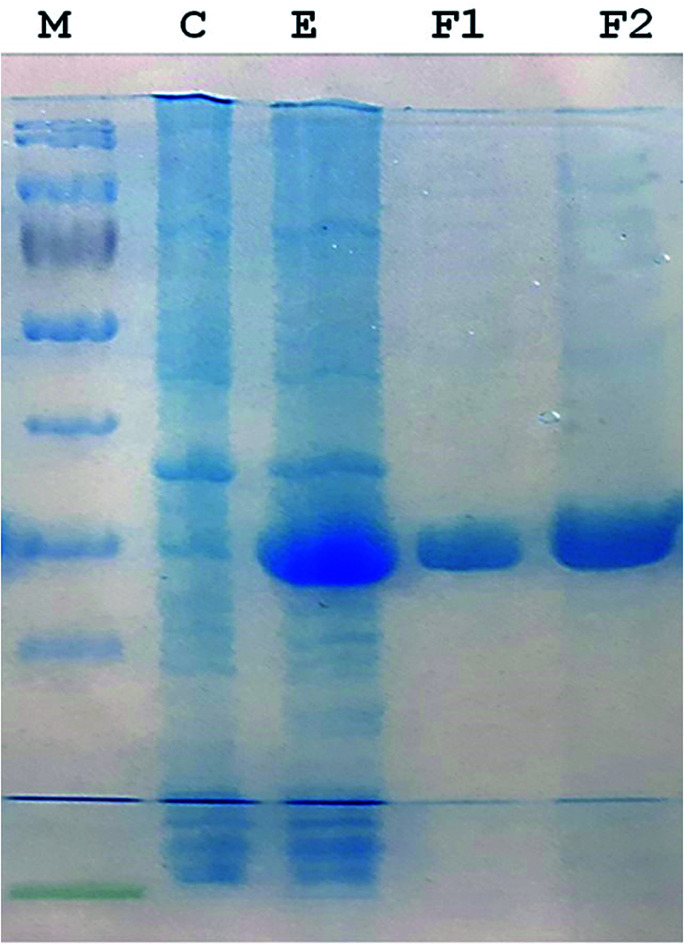

## Introduction

l-Glutaminase (E.C.3.5.1.2) is l-glutamine amidohydrolase, which catalyzes the conversion of glutamine to glutamic acid by the release of ammonia. l-Glutamine is a non-essential amino acid found in high concentrations in the blood, and it serves as the primary fuel for the rapid growth of cancer cells.^1,2^ In fact, majority of the tumour cells exhibit glutamine addiction, and under hypoxic conditions, their glutamine consumption is 15 times higher than that of the normal cells.^3^ Although cancer cells use glutamine in large amounts, they are unable to produce their own glutamine *de novo*.^1,4^ To be well used by cancer cells, glutamine is made available through specific transporters, and it must be converted into glutamate by the activity of glutaminase. Many glutaminase inhibitory molecules have shown anticancer activity by inducing apoptosis.^5,6^ Thus, l-glutaminase is an attractive target to block the energy route for the proliferation of glutamine-dependent tumour cells and responsible for their selective death.^7,8^ Several other applications of the enzyme have been reported due to its effective antiretroviral activity;^9^ moreover, the enzyme is used to improve the taste and aroma of the food products.^10^l-Glutaminase also has a significant application in biosensors where it is used to monitor the level of glutamine in mammalian and hybridoma cells.^11,12^ In the case of fermented foods, l-glutaminase is widely used to enhance the taste and aroma. In fermented food products, such as miso, soy sauce and sufu, the enzyme increases the content of glutamic acid, which provides a pleasant taste.^13,14^ The wide range of applications of glutaminase has initiated a quest for searching a more stable and novel enzyme that can be exploited after large-scale production. The enzymes obtained from thermophillic microorganisms have better stability and activity at industrial temperatures.^15,16^ Modern tools and techniques of genetic engineering have made it possible to produce the recombinants of useful enzymes and proteins in modified prokaryotic gene expression systems. Recombinants of glutaminases have been produced from different sources including *Pseudomonas nitroreducens*,^17^*Micrococcus luteus*,^18^ and rat kidney.^19^ The recombinant enzyme has been purified and characterized for its physiochemical activities and potential applications. In the present study, we performed cloning, expression and characterization of l-glutaminase obtained from *Geobacillus thermodenitrificans* DSM 465. A comparative structural analysis of human and *Geobacillus* enzymes was made. The binding affinities of different inhibitory molecules with the enzyme were determined by *in silico* approach.

## Materials and methods

### Reagents and kits for DNA manipulations

Genomic DNA of *Geobacillus thermodenitrificans* DSM-465, used as a template for the amplification of glutaminase gene, was purchased from DSMZ, Germany. *E. coli* strain BL21 (DE3) RIL codon plus, and cloning and expression vectors pTZ57R/T and pET21a (+) were obtained from ThermoFisher and Novagen. DNA marker, 1 kb DNA Ladder (#SM0311), and prestained protein marker PAGERuler™, 10–180 kDa (26616), were purchased from Thermo Fisher Scientific (USA). DNA restriction enzymes (*Nde*I and *Bam*HI), InsTAclon PCR Cloning Kit (#1213), PCR reagents and *Taq* DNA polymerase were obtained from Thermo Fisher Scientific (USA). The Gel DNA extraction kit GenElute™ Gel Extraction Kit (NA1111) was purchased from Sigma-Aldrich (USA). All routine laboratory chemicals were of analytical grade.

### PCR amplification and cloning of glutaminase gene

*Geobacillus thermodenitrificans* DSM-465 glutaminase gene comprises 930 bp open reading frame (Accession no. NC 009328). Forward and reverse primers with 5′-catatggggtttcaaggtaacggtgtcac-3′ and 5′-atggatccttaagccggacgcaaatg-3′ were used for PCR amplification of gene. The reaction mixture containing 2.5 mM MgCl_2_, 2.0 mM dNTPs, 40 picomole primers, 15–20 ng of template DNA, and 2.5 U *Taq* DNA polymerase was subjected to thermocycler conditions of 94 °C, 63 °C and 72 °C for 35 cycles. The PCR-amplified glutaminase gene was analysed *via* agarose gel electrophoresis and purified using the GenElute™ Gel Extraction Kit (Sigma-Aldrich) according to the procedure described by the supplier. The purified PCR product was ligated into pTZ57R/T cloning vector; ligation mixture was incubated overnight at 22 °C and used for the transformation of bacterial cells. The TransformAid bacterial transformation kit (Thermo Fisher Scientific, catalogue no. k2710) was used for the transformation of bacterial competent cells with the recombinant plasmid. The successfully transformed colonies were screened for the presence of glutaminase gene by isolation and restriction of plasmids with *Nde*I and *Bam*HI followed by agarose gel electrophoresis. The gene was subcloned in the pET21a (+) plasmid, and recombinant *E. coli* cells were obtained by plasmid isolation and restriction analysis, as abovementioned.

### Production and purification of recombinant glutaminase

To produce recombinant glutaminase, ampicillin-containing LB broth was inoculated with a single confirmed colony in a flask, and the flask was incubated overnight at 37 °C and 200 rpm in an orbital shaker. After this, one percent of the overnight culture was transferred to ampicillin-containing LB broth and grown under the abovementioned conditions to obtain an optical density of 0.3 at 600 nm. Cells were regularly monitored for their growth by measuring the OD of the culture at 600 nm. The protein production was induced overnight during incubation in the presence of 0.2 mM IPTG at 20 °C and 100 rpm. The control cells (transformed with the pET21a (+) lacking glutaminase gene) were also processed in parallel. The cells were obtained by centrifugation at 10 000 × *g* for 5 min, and the pellet was suspended in distilled water and analysed *via* SDS-PAGE with a protein marker.

### Measurement of the glutaminase activity

The activity of glutaminase was determined by the modified Nesslerization method adopted from the literature.^7^ Briefly, 2800 μL reaction mixture contained 3.5 mM of glutamine solution prepared in 50 mM Tris–HCl buffer, pH 9.0. To this mixture, 100 μL (100 μg) of the enzyme was added, and the mixture was incubated for 10 min at 70 °C. The reaction was terminated by the addition of 100 μL of 500 mM trichloroacetic acid after the incubation period. Nessler's reagent (500 μL) was added to the reaction mixture, which was then mixed well and incubated for 10 min. The absorbance was measured at 480 nm. Herein, one unit of enzymatic activity was defined as the amount of enzyme required to release one μ mol of ammonia per mL per min.

### Enzyme kinetics

The effect of temperature on the enzyme activity was determined by adjusting the temperature of the reaction mixture from 40 °C to 100 °C. The optimum pH for enzyme activity was determined by preparing the reaction mixture in the buffer solutions adjusted at different pH values ranging from 6 to 12. The effect of pH on the enzyme activity was measured at 70 °C. The buffer solutions were selected on the basis of their p*K*_a_ values and effective buffering range. The influence of pH was determined using phosphate buffer adjusted at the pH values of 6, 6.5, 7 and 7.5 followed by Tris–HCl buffer adjusted at the pH values of 8, 8.5, 9, 9.5,…12. The effect of glutamine concentration on the activity of glutaminase was determined, and the *K*_M_ and *V*_max_ values were calculated by the Lineweaver–Burk plot.

### Protein modelling and validation

Since the protein structure of GLS-GT (glutaminase obtained from *Geobacillus thermodenitrificans* DSM-465) was unavailable at the PDB server, we generated its 3D model by Swiss-Model^20^ and I-TASSER^21^ servers *via* employing the crystal structure ‘2PBYa’ (GLS protein of *Geobacillus kaustophilus* HTA426 that share ∼90% similar sequence) as the template. Subsequently, the quality of the predicted model was examined by RAMPAGE, a protein structure validation server,^22^ which revealed the results in terms of phi, psi and Cbeta deviations by generating a Ramachandran plot for the protein built. The quaternary structure analysis was performed by QSQE, which is a quaternary structure prediction algorithm server.^23^

### Structural alignment

The TM-align server was used to check the structural similarity of the GLS-GT with other proteins in the databases. TM-align is an algorithm for sequence-order-independent protein structure comparisons that generate optimized residue-to-residue alignment based on structural equivalence using dynamic programming iterations for two protein structures of unknown similarity. As an output, an optimal superimposition of the two structures and the TM-score values scaling the structural similarity were retrieved. The TM-score value ranges between 0 and 1, where 1 indicates a perfect match between the two structures.^24^ Moreover, GLS-GT and GLSK (227–533) were super-imposed by PyMOL for visual analysis; ConSurf web server^25^ identified and compared the evolutionary conserved residues.

### Molecular docking and comparative analysis

3-D structures of the selected potential inhibitors were retrieved from the chemical structure databases, such as PubChem and ChemSpider servers, and subjected to molecular docking against the GLS-GT protein using the Hex docking server.^26^ The Δ*G* (binding free energy) of each docked protein-inhibitor complex was determined to rank the strongest inhibitor. The same set of inhibitors was analysed against the human GLS-K (kidney isoform) (3CZD) protein to have a comparative analysis. Initial inhibitors present in the 3CZD complex structure were removed manually to avoid docking error with desired selected inhibitors. The details of the inhibitory molecules investigated in the present study are provided in Table 2.

## Results

### Gene cloning and expression

**Fig. 1:**
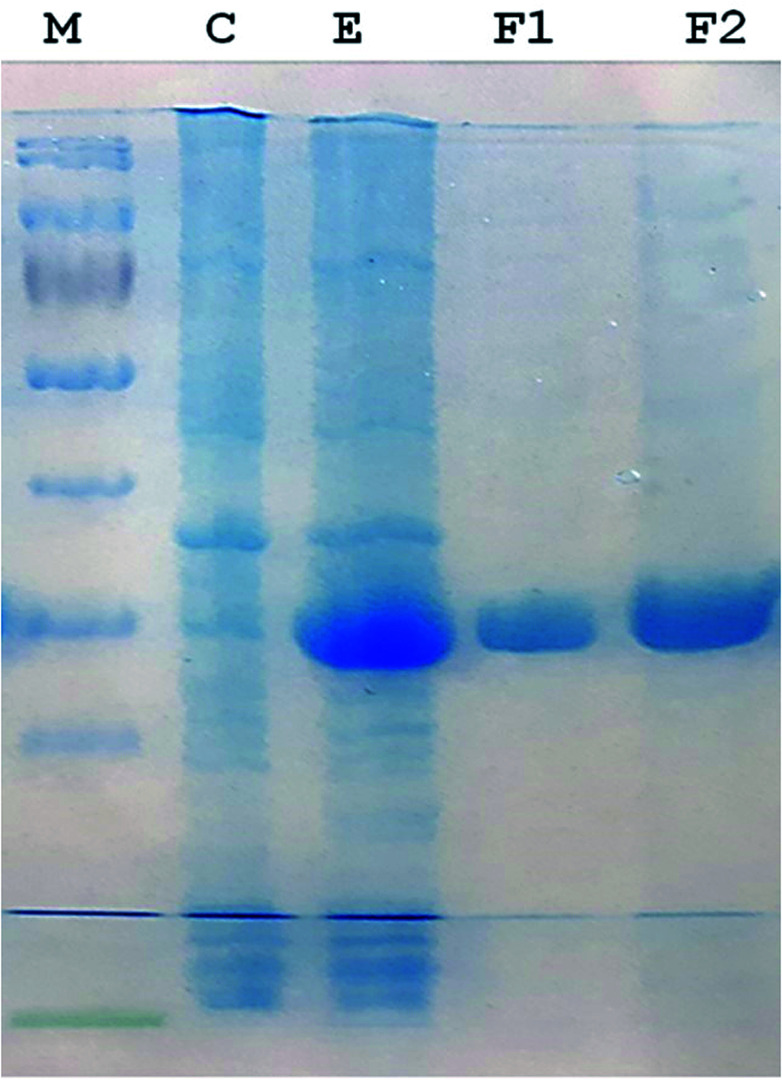
SDS-PAGE (12%) image. (M) Protein marker (Thermo Fisher Scientific PageRulerTM catalogue no. 26616). (C) Protein pattern of *E. coli* cells transformed with pET 21a (+) (plasmid lacking DNA encoding glutaminase). (E) Recombinant glutaminase expressed in *E. coli* transformed with recombinant pET-GT. (F1 and F2) Fractions of purified recombinant glutaminase after anion exchange chromatography.

A 930-bp DNA fragment representing the glutaminase gene was PCR amplified, cloned into pTZ57R/T and subcloned in pET21a (+) plasmids. The cells transformed with the recombinant pET-GT plasmid were subjected to gene expression, which provided a prominent protein band at about 33 kDa *via* SDS-PAGE. The recombinant protein was purified up to electrophoretic homogeniety (Fig. 1).

### Purification and kinetics of recombinant glutaminase

**Fig. 2:**
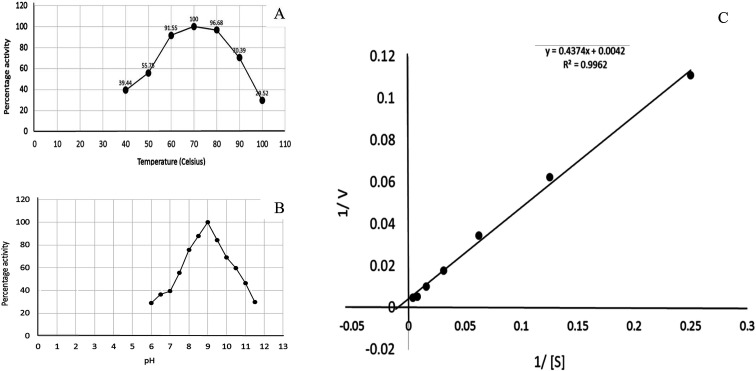
Kinetics of recombinant glutaminase. (A) Effect of temperature on the enzyme activity, (B) effect of pH on the enzyme activity and (C) Lineweaver–Burk plot for the calculation of *K*_M_ and *V*_max_.

Recombinant glutaminase was purified by DEAE-cellulose-based anion-exchange chromatography. Phosphate buffer (20 mM, pH 7.5) was selected as the mobile phase, the sample was applied on to the column at the 2 mL per minute flow rate, and protein was eluted by a linear NaCl gradient from 0 to 0.5 M. Fractions comprising 3 mL of elution sample were obtained and analysed for enzyme activity *via* SDS-PAGE (Fig. 1). The fractions with purified enzyme were pooled together. The specific activity of purified enzyme was 138 units per mg of enzyme, and total 8700 units of purified enzyme were obtained with 40.4% recovery and 22.36-fold purification (Table 1). Optimum enzyme activity was measured at 70 °C and pH 9; the *K*_M_ value for l-glutamine was 104 μM, and the *V*_max_ value for enzyme was found to be 238.1 μmol per min (Fig. 2).

**Table 1:** Activity, total units, total protein content, specific activity, percentage recovery and fold purification of recombinant l-glutaminase at different purification stages

Purification step	Activity U mL^−1^	Protein mg mL^−1^	Specific activity	Total units	Percentage recovery	Fold purification
Crude supernatant	105	17	6.17	21 500	100	1.00
Acetone precipitation	197	10	19.70	17 800	82.7	3.2
DEAE-cellulose column	290	2.10	138.0	8700	40.4	22.36

### Protein modelling and validation

**Fig. 3:**
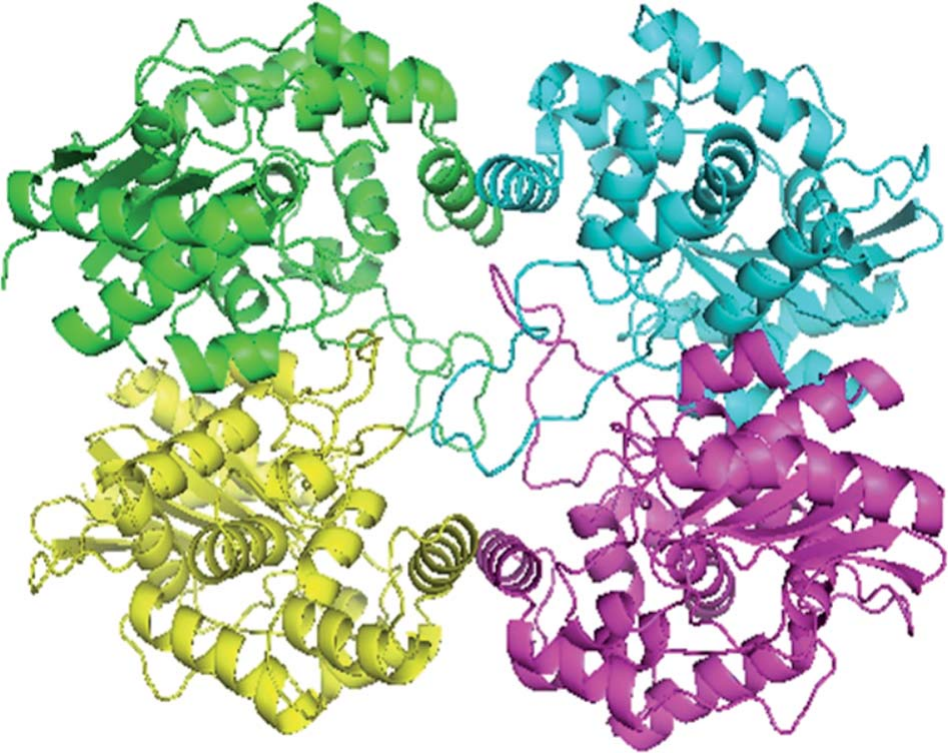
3-D model of GLS-GT (*Geobacillus thermodenitrificans* DSM-465) enzyme in its tetramer form represented as transparent surface mode with cartoons. Chain A: green; chain B: cyan; chain C: magenta; chain D: yellow (visualized by PyMol).

Swiss-Model generated the GLS-GT homo-tetramer models *via* the QSQE server. Homotetramer, visualized by PyMOL (Fig. 3), was opted for our further study. Molecular Graphics System, version 1.2r3pre, collectively reveal that the template-based structure of the GLS-GT built has excellent quality and stability; this is perhaps due to the fact that the template crystal model (2PBY) contains nearly the same number of residues (306) in its protein sequence, which are structurally >88% identical to those of GLS-GT. The quaternary structure of GLS-GT was validated to be a homotetramer through the structure clustering tree of GLS-GT homologs with other known structures (ESI Fig. SF1 and SF2; ESI Table ST1†).

### Structural alignment report

**Fig. 4:**
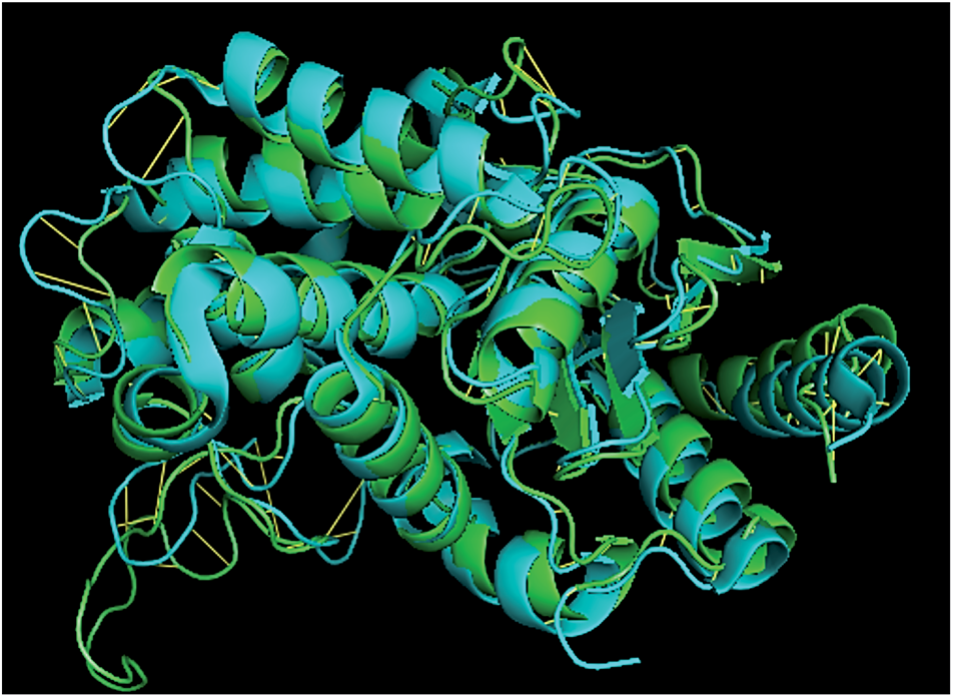
GLS-GT monomer (green) and GLSK monomer (cyan) superimposed for structural analysis.

TM-align results have predicted that 1u60A (Glutaminase protein of *Escherichia coli*-K12) is structurally most similar to GLS-GT, whereas the human glutaminase kidney isoform (GLS-K) ranks second in structural similarity. However, both the structures (GT and human enzymes) have less than 40 percent sequence identity in the structurally aligned region. The structural superimposition of GLS-K and GLS-GT monomers has shown more than 90% identity (Fig. 4; ESI Table ST2†).

### Docking and comparative analysis

**Fig. 5:**
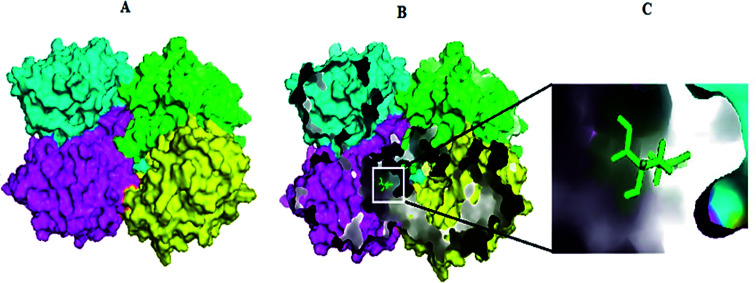
Molecular docking complex exhibiting GLS-GT homotetramer chains in surface view and the substrate (glutamine-GLN) in sticks. (A) Docked complex of GLS-GT and GLN. (B) Internal pockets mode visualizing docked complex of GLS-GT and GLN. (C) Zoom-in view of GLN docked with GLS-GT. Chain A: green; chain B: cyan; chain C: magenta; chain D: yellow.

**Fig. 6:**
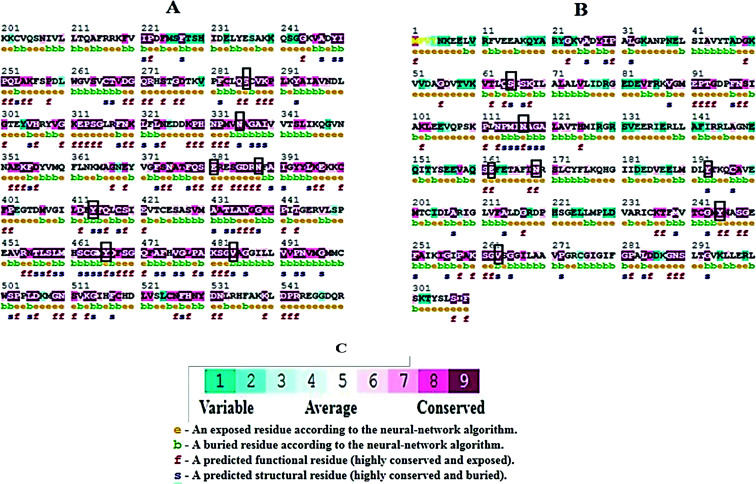
ConSurf output showing conserved regions in maroon. (A) GLS-K human partial protein sequence (200aa–550aa) (B) GLS-GT protein sequence. Black boxes highlight the active-sites similarity of both proteins; (e) exposed residues; (b) buried residues; (f) functional residues; (s) structural residues. (C) Color-coded conservation scale of ConSurf output.

Molecular docking results of glutamine with the enzyme active site have been described in the present study (Fig. 5). Docking results have also revealed the binding energies of both protein (GLS from *Geobacillus thermodenitrificans* and *Homo sapiens*) when they are bound with some candidate inhibitors (Table 2). The structural comparison by superimposition reveals that although the sequences of GLS-GT and GLSK are less than 40% similar, the GLS-GT monomer is 94% structurally identical to the GLSK monomer (3CZDA). ConSurf webserver exhibited the conservation, exposed and buried states of the residues. The continuous conservation scores are divided into a discrete scale of nine grades for visualization, from the most variable positions grading 1 (turquoise), through intermediately conserved positions grading 5 (white), to the most conserved positions grading 9 (maroon) (Fig. 6). In addition to majority of structural domains, the active site residues were found to be conserved among glutaminase obtained from the subject bacterial strain and the corresponding human enzyme.

**Table 2:** Candidate inhibitory molecules for glutaminase, and a comparison of glutaminase from *Geobacillus thermodenitrificans* with kidney-type glutaminase (GLS-K) from *Homo sapiens* on the basis of free energy changes calculated by docking analysis

Enzyme	Inhibitory molecule	Δ*G* (kJ mol^−1^)	Rank	Enzyme	Inhibitory molecule	Δ*G* (kJ mol^−1^)
GLS-GT	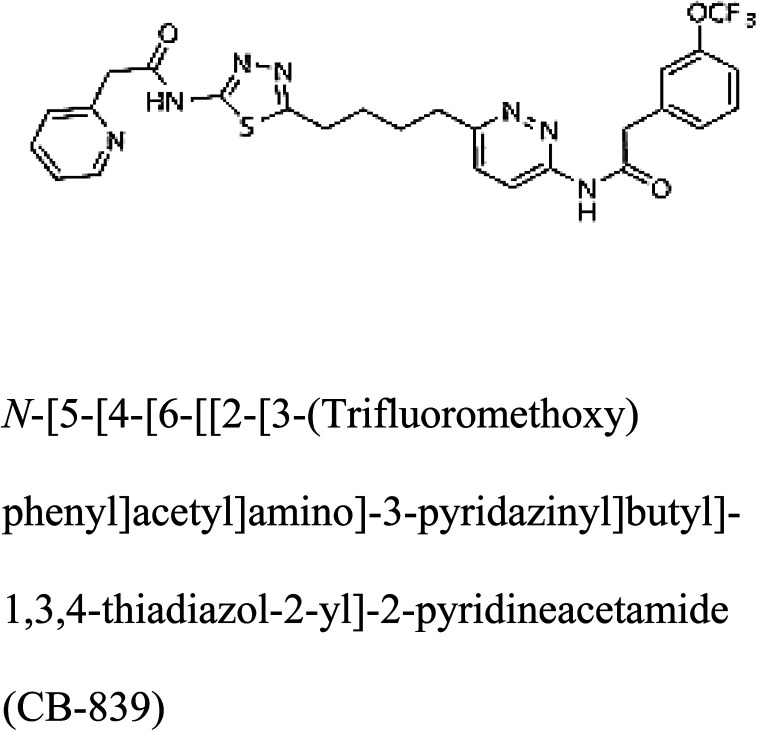	−388.7	1	GLS-K	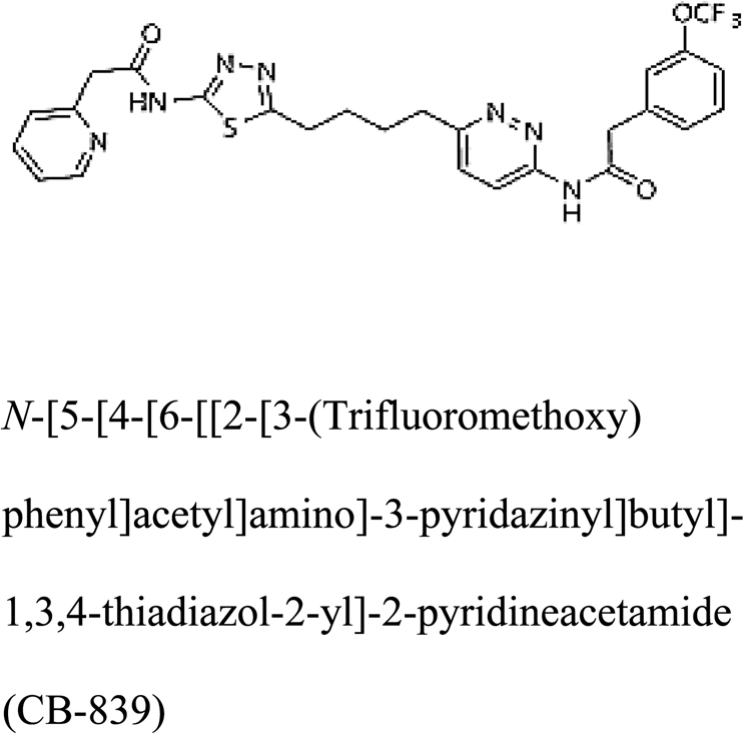	−359.16
	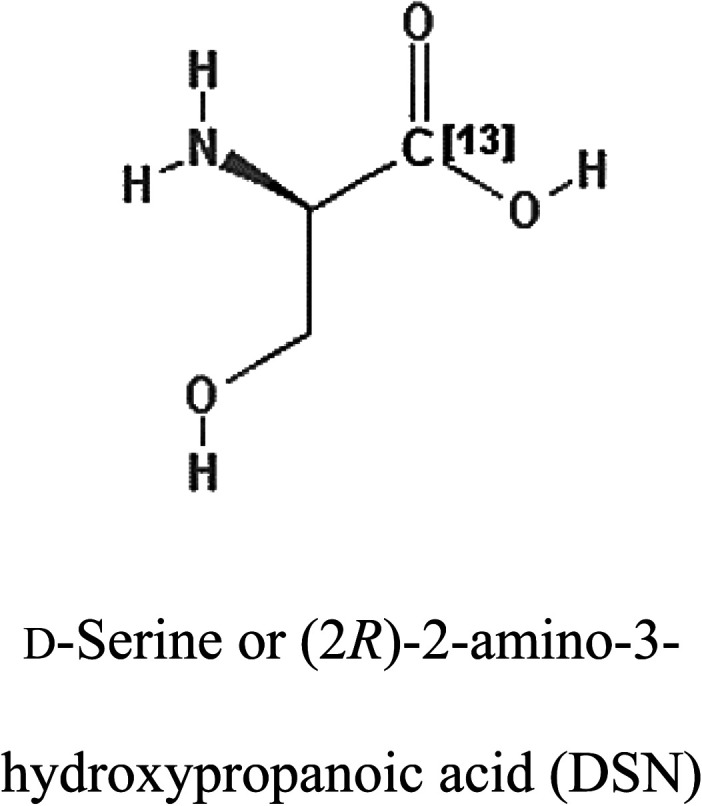	−149.67	2		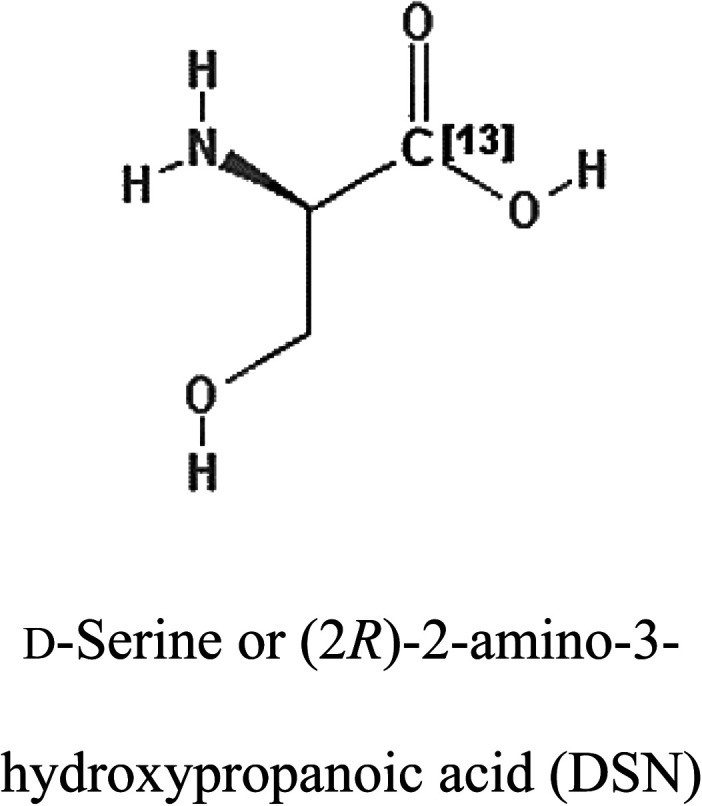	−150.7
	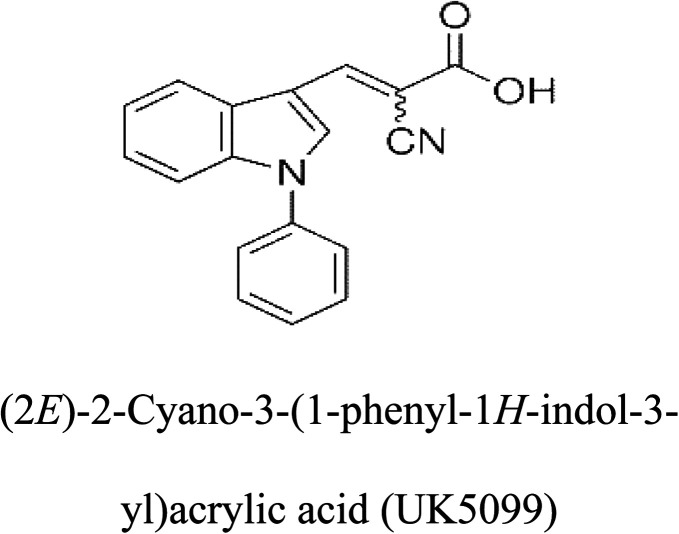	−265.06	4		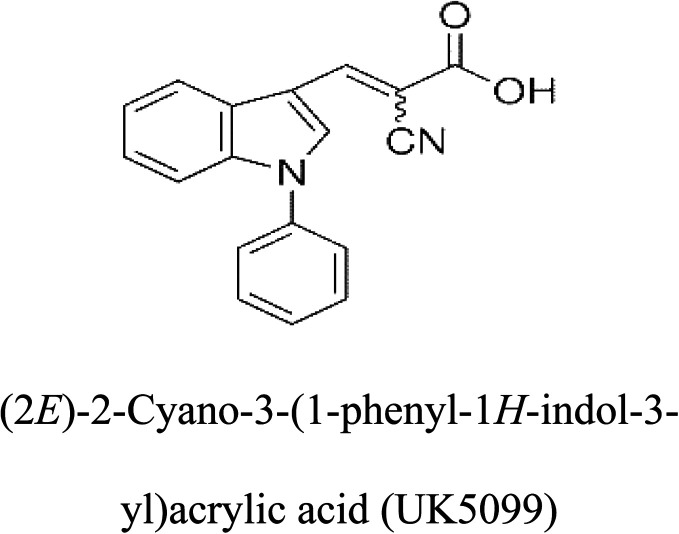	−260.7
	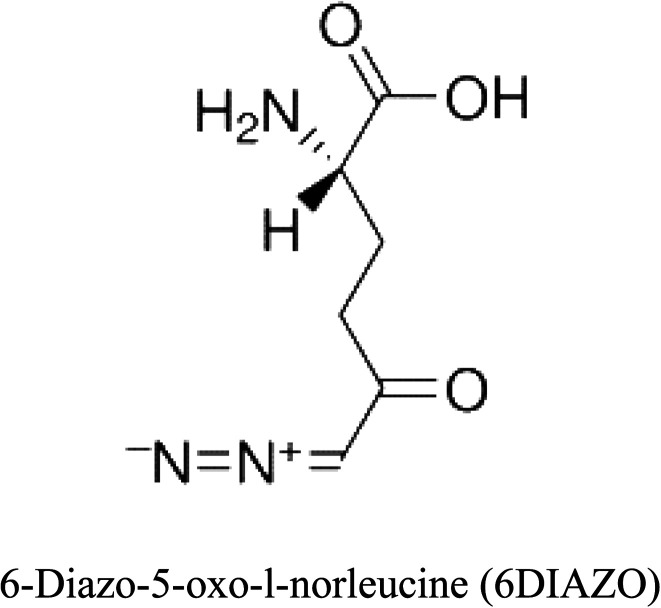	−195.42	4		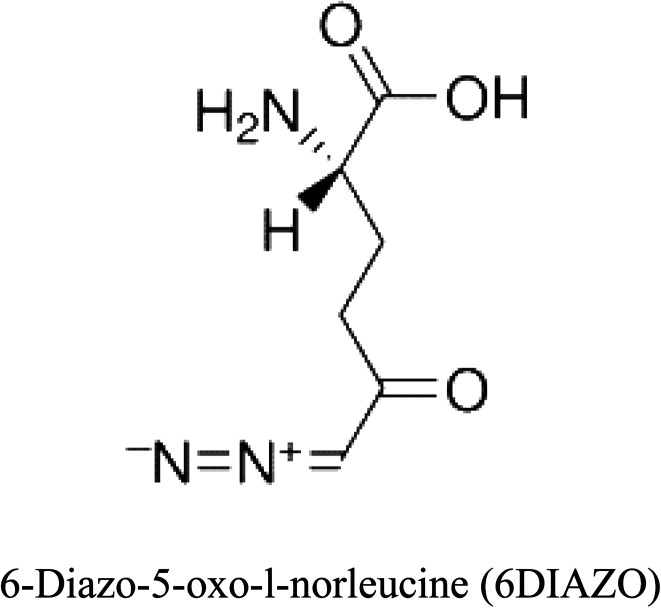	−178.21
	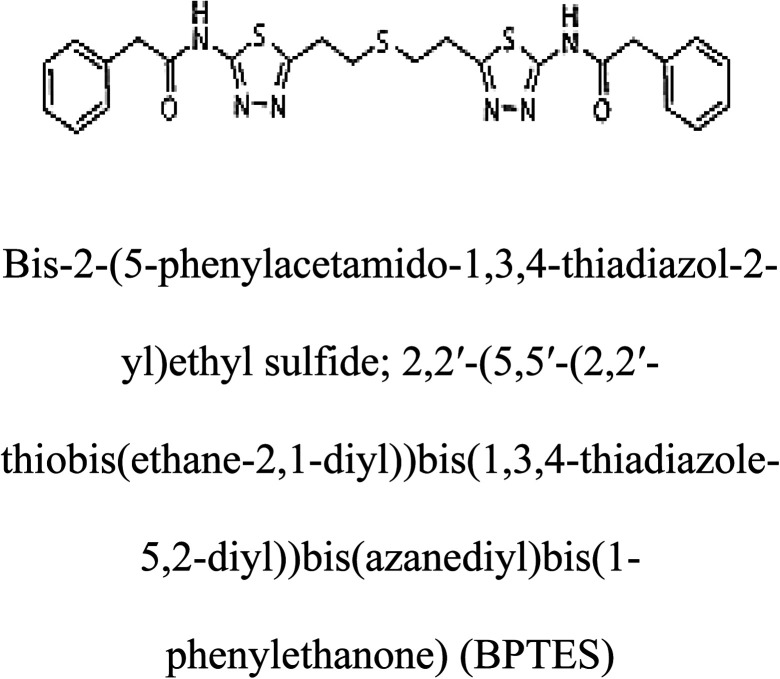	−62.15	5		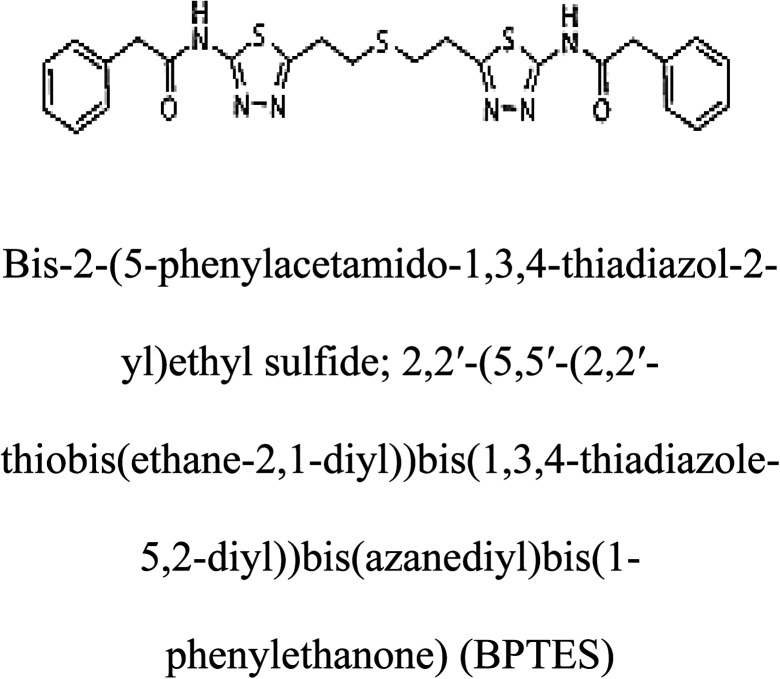	−356.99
	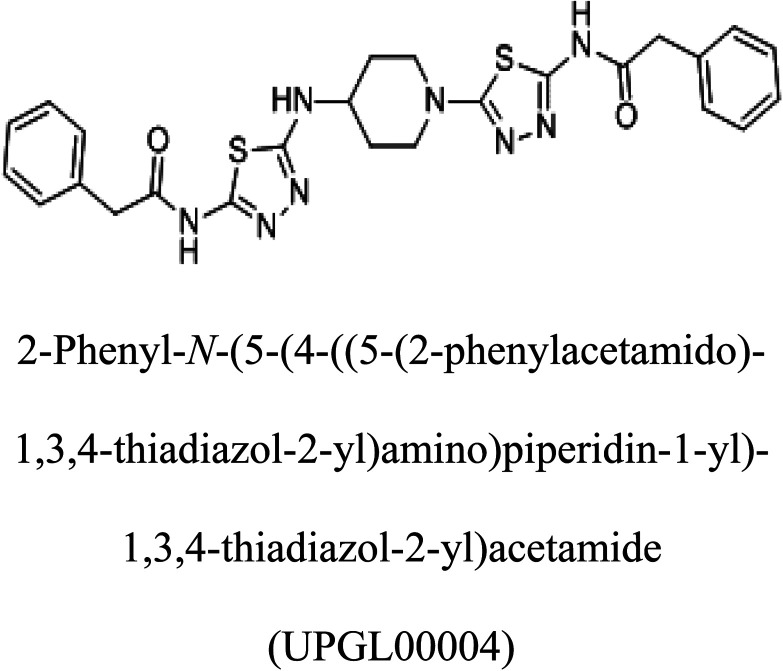	−44.56	6		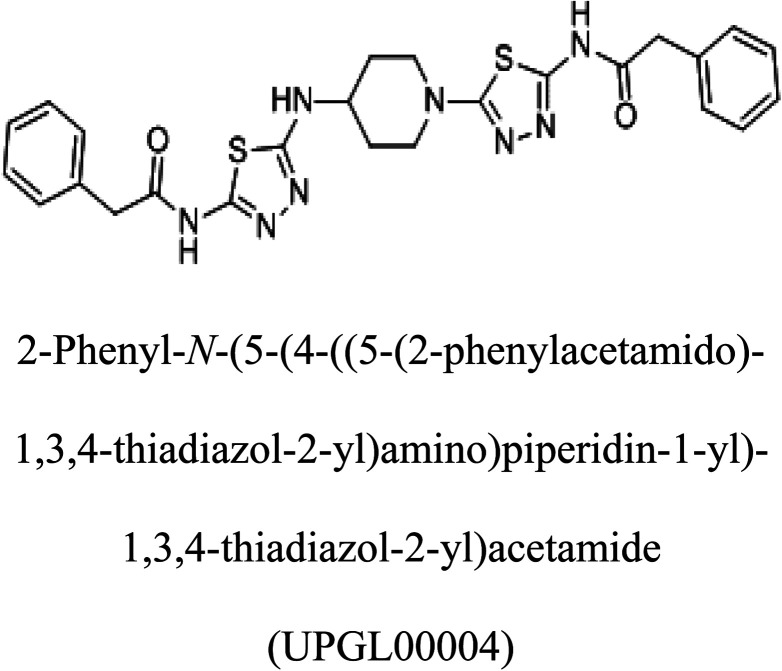	−375

## Discussion

Glutamine is the major plasma amino acid that serves as a major energy source for cancer cells. Glutamine is converted to glutamate by the activity of glutaminase I, which enters the TCA cycle as α 2-KG. Kidney-type glutaminase (glutaminase 1) is, therefore, overexpressed in majority of cancer cells as an indicator of cancer prognosis,^27^ and its inhibition serves as a vital anticancer strategy.^28,29^ Withdrawal of plasma glutamine or inhibition of glutaminase activity halts the growth of cancer cells in mouse models.^30,31^ Studies on the conserved protein structural domains and their active site pockets from different species can lead to the identification of more potent and versatile inhibitors.^32,33^ In the present study, we have produced a recombinant of glutaminase from *Geobacillus thermodenitrificans* DSM-465 in *E. coli*. The purified glutaminase exhibited a molecular weight of 33 kDa *via* SDS-PAGE (Fig. 1). The enzyme obtained from *Streptomyces canarius* has a molecular weight of 44 kDa,^8^ that obtained from *Rhizobium etli* has a molecular weight of 26.9 kDa,^34^ and that obtained from *Pseudomonas nitroreducens* has a molecular weight of 40 kDa.^35^ The recombinant enzyme has shown optimum activity at 70 °C and pH 9. No activity was observed at pH below 5. The *K*_M_ value for glutamine was 104 μM (Fig. 2). The specific activity of enzyme was 138 U per min per mg of protein, and the enzyme was purified up to 22 folds, with 40% recovery (Table 1). The comparison of kinetic properties of GSL-GT with those of human GSL-K^36^ has shown a close pH for optimum activity (pH 9 for GSL-GT and pH 8.6 for GSL-K) in the Tris buffer. However, the bacterial enzyme exhibited maximum activity at a much higher temperature of 70 °C as compared to GSL-K (37 °C). The human enzyme has a *K*_M_ value of 22.99 mM, and the bacterial recombinant enzyme exhibited a *K*_M_ value of 140 μM for l-glutamine; this indicated a better substrate affinity of bacterial enzyme. These results indicate moderate to high thermostability of enzyme with better affinity to glutamine as compared to the previous reports.^7,8^ A 3-D model of glutaminase obtained from *Geobacillus thermodenitrificans* DSM-465 was built using the QSQE server,^23^ which appeared as a homotetramer when visualized by PyMOL (Fig. 3). The 3-D structure was validated by the Molecular Graphics System, version 1.2r3pre, and it was found to be a very good quality protein model (Fig. SF1 and SF2; Table ST1†). These computer-based studies have been recently conducted to determine the structural basis of enzyme activity.^37^ A comparative analysis of our subject enzyme with human glutaminase I indicated only 40% homology in the amino acid sequence. However, the superimposed 3-D models of both enzymes have shown up to 90% identity in the secondary and tertiary structures (Fig. 4; ESI Table ST2†). The results indicated conservation of their secondary structures despite the variable primary structure of proteins that might be adaptations during evolution. The docking complex of recombinant glutaminase was prepared with its substrate (glutamine), and the binding pocket for the substrate was determined (Fig. 5). ConSurf output was used to determine the evolutionarily conserved structural and active site regions in human and subject bacterial glutaminases.^38^ The results have indicated the conservation of putative active site residues of GLS-GT at the amino-acid positions Ser65, Asn117, Glu162, Asn169, Tyr193, Tyr245, and Val263 (Fig. 6) that have been found to be conserved at the active site of the human GLS-K enzyme, *i.e.* Ser286, Asn335, Glu381, Asn388, Tyr414, Tyr466 and Val484 amino-acid residues.^39^ Another study has recently revealed that Tyr446 and Val484 of GLS-K play a significant role in the ligand (physapubescin) binding by π–π stacking interaction and hydrophobic interaction, respectively,^40^ supporting the predicted ligand binding role of Try245 and Val263 (GSL-GT) in the current study.

In addition to the active site amino acids, several other protein domains were also found to be conserved when partial protein sequence of human GLS-K and GLS-GT was considered. The conservation-based color-coding result reveals that all the active sites are highly conserved across the diverse species. Molecular docking of GLS-K and GLS-GT with six potential inhibitory molecules has shown a variable degree of binding affinity between the enzyme active site and inhibitory molecules, as indicated by the binding free energy change (Δ*G*) values (Table 2). According to our calculations, CB-839 was the best inhibitor for GLS-GT and UPGL00004 was the best inhibitor for GLS-K, as indicated by the binding free energy changes, *i.e.* Δ*G* −388.7 kJ mol^−1^ and Δ*G* −375 kJ mol^−1^, respectively. There are various reports on the reliability of calculations made on the basis of molecular docking studies, suggesting 30–37% probability of correct measurement.^41,42^ However, the docking-based affinity calculations can be applied to screen a large number of potential protein partner molecules, saving a huge amount of money, time and efforts in the laboratories. Our findings correlate with the recent reports where CB-839 and UPGL00004 have been shown as the most potent GLS inhibitors;^43^ this indicates the validity of our findings. The inhibitor molecules have been graded according to their potential efficacy against the bacterial and human glutaminase as suggested by the binding free energy changes (Table 2) calculated by the molecular docking experiments. The findings can be used for X-ray/NMR structure analysis in *in vitro* and *in vivo* anticancer studies.

## Conflicts of interest

The authors declare no conflict of interest.

## Supplementary Material

RA-009-C8RA04740E-s001
