# Supplementary material for: Recombinant l-glutaminase obtained from Geobacillus thermodenitrificans DSM-465: characterization and in silico elucidation of conserved structural domains
Source: RSC Adv. 2019 Feb 1;9(8):4258–67. doi: 10.1039/c8ra04740e (PMC9060542; doi:10.1039/c8ra04740e)
Supplement: RA-009-C8RA04740E-s001 [file RA-009-C8RA04740E-s001.pdf]

Electronic Supplementary Material (ESI) for RSC Advances.
This journal is © The Royal Society of Chemistry 2019

SUPPLEMENTARY DATA

Table ST1. The validation of protein 3-D model using various parameters

Protein	C-score[a]	TM-score[b]	RMSD[c]	IDEN[d]	Cov[e]
GLS-GT	1.72	0.96	2.8Å	0.889	0.996

[a] Confidence (C) score for estimating the quality of predicted models (range -5 to 2].

[b] Template Modeling Score (TM-score) measure the structural similarity between two structures TM-score >0.5 indicates a model of correct topology and a TM-score<0.17 means a random similarity range 0 to 1).

[c] Heavy atoms Root-Mean-Square Deviation (RMSD) with respect to the experimental structure.

[d] IDEN is the percentage sequence identity in the structurally aligned region (range 0 to 1

[e] Cov represents the coverage of global structural alignment and is equal to the number of structurally aligned residues divided by length of the query protein cluster. (range 0 to 1)

Table ST2. Structural alignment report by TM-align, representing the rankwise PDB hits. 2nd rank bold highlighted is the report of comparison with GLS-K human. 5th rank 2pbyA is observed with highest sequence similarity (89%) in the structurally aligned region.

**Rank**	**PDB Hit**	**TM-score**	**RMSD[a]**	**IDEN[a]**	**Cov**
1	1u60A	0.982	0.63	0.389	0.990
**2**	**3czdA**	**0.948**	**1.18**	**0.358**	**0.977**
3	3ihbA	0.943	1.57	0.397	0.987
4	5uqeA	0.941	1.67	0.361	0.987
5	**2pbyA**	0.900	1.51	**0.890**	0.942
6	1mkiA	0.885	1.56	0.474	0.926
7	2wadC	0.699	3.72	0.123	0.851
8	2wafA	0.696	3.68	0.111	0.845
9	3vskA	0.694	3.75	0.094	0.845
10	3fwmA	0.681	3.18	0.119	0.796

- Ranking of proteins is based on TM-score of the structural alignment between the query structure and known structures in the PDB library.
- RMSD[a] is the RMSD between residues that are structurally aligned by TM-align.
- IDEN[a] is the percentage sequence identity in the structurally aligned region.
- Cov represents the coverage of the alignment by TM-align and is equal to the number of structurally aligned residues divided by length of the query protein

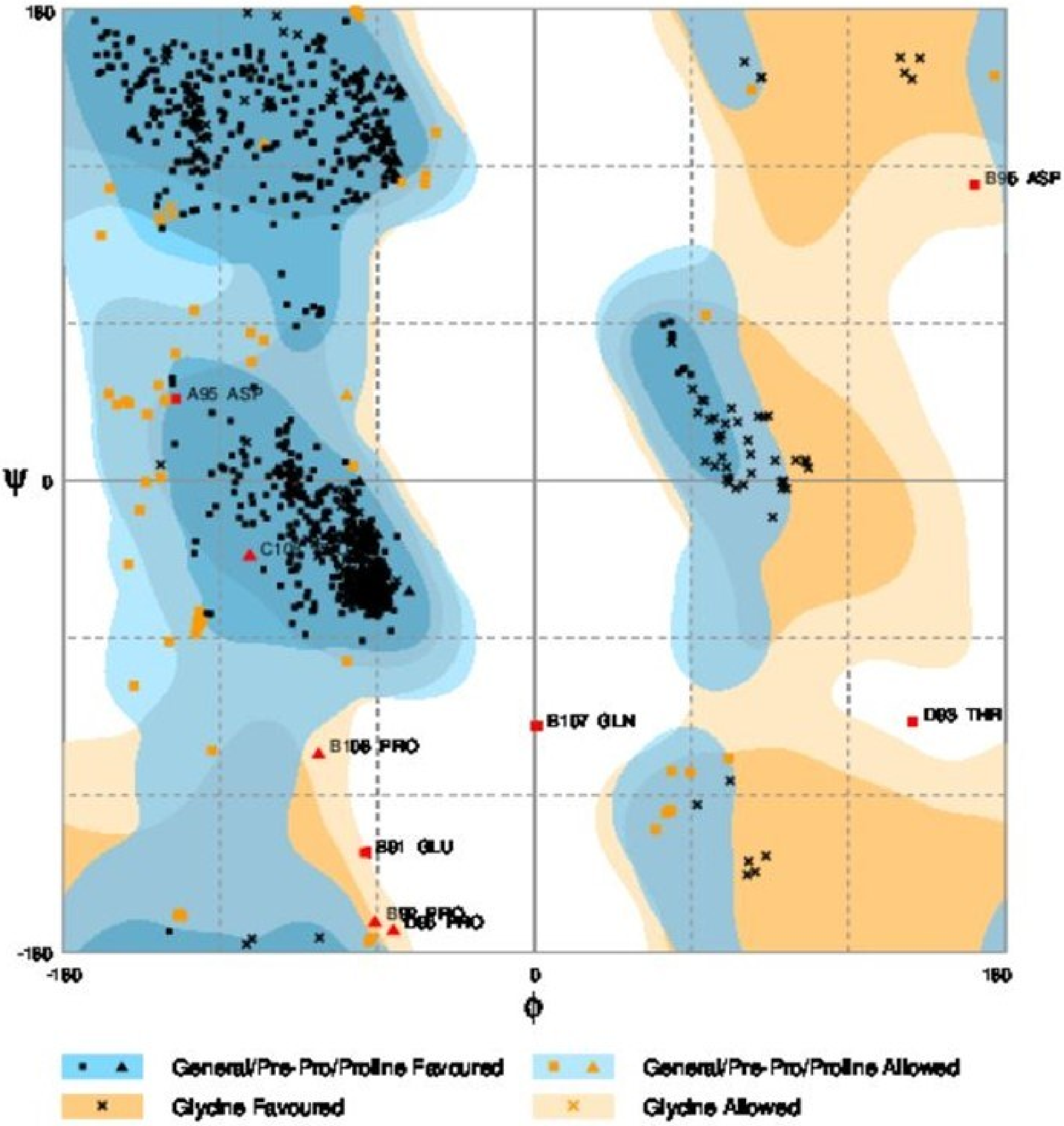

Figure SF1. The Ramachandran Plot generated by the RAMPAGE server depicting the quality of the built GLS-GT protein.

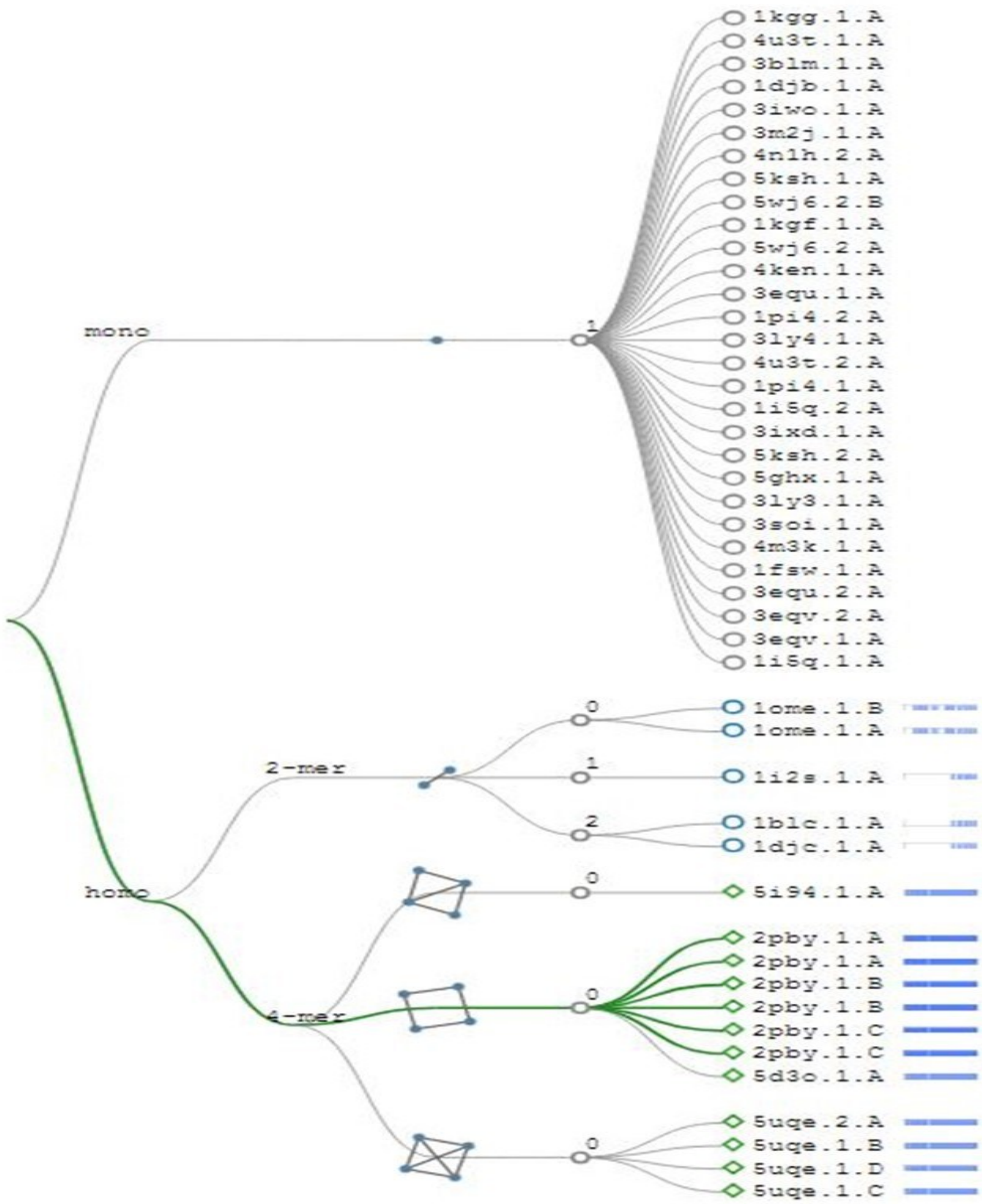

Figure SF2. Quaternary structure analysis report predicted by QSQE. Structural clustering tree of GLS-GT homologs with other known structures. Each leaf is a template labelled with the PDB code and a bar indicating sequence identity and coverage (darker shades of blue refer to higher sequence identity). The green thread indicates templates with predicted conserved quaternary structures.
